# Sitagliptin Mitigates Diabetic Cardiomyopathy Through Oxidative Stress Reduction and Suppression of VEGF and FLT-1 Expression in Rats

**DOI:** 10.3390/biom15081104

**Published:** 2025-07-30

**Authors:** Qamraa H. Alqahtani, Tahani A. ALMatrafi, Amira M. Badr, Sumayya A. Alturaif, Raeesa Mohammed, Abdulaziz Siyal, Iman H. Hasan

**Affiliations:** 1 Department of Pharmacology and Toxicology, College of Pharmacy, King Saud University, P.O. Box 22452, Riyadh 11495, Saudi Arabia; ghamad@ksu.edu.sa (Q.H.A.); amibadr@ksu.edu.sa (A.M.B.); 43204515@ksu.edu.sa (S.A.A.); 2 Department of Anatomy, College of Medicine, King Saud University, P.O. Box 2925, Riyadh 11461, Saudi Arabia; talmatrafi@ksu.edu.sa; 3 Department of Histology, College of Medicine, King Saud University, P.O. Box 2925, Riyadh 11461, Saudi Arabia; rmohammad@ksu.edu.sa (R.M.); asiyal@ksu.edu.sa (A.S.)

**Keywords:** diabetes mellitus, myocardial fibrosis, oxidative stress, cardiovascular disease, Sitagliptin

## Abstract

Diabetes mellitus (DM) is a global health challenge marked by chronic hyperglycemia, which can result in complications such as diabetic cardiomyopathy. Sitagliptin, an oral anti-hyperglycemic drug, has demonstrated efficacy in alleviating cardiovascular complications associated with DM. This study explored the impact of Sitagliptin’s potential as a therapeutic agent, functioning not only to control blood sugar levels but also to enhance vascular health and strengthen cardiac resilience in diabetes. The investigation focused on alterations in the vascular endothelial growth factor (VEGF) and its receptor-1 (FLT-1) signaling pathways, as well as its potential to suppress inflammation and oxidative stress. A number of rats received a single dose of streptozotocin (STZ) 55 mg/kg (i.p.) to induce DM. Sitagliptin was administered orally (100 mg/kg/90 days) to normal and diabetic rats, after which samples were collected for investigation. Sitagliptin significantly mitigated weight loss in diabetic rats. Its administration significantly reduced blood glucose levels and improved serum troponin I and CK-MB levels. Heart sections from diabetic rats showed a marked increase in mTOR, VEGF, and FLT-1 immune reaction, while sitagliptin-treated diabetic rats’ heart sections showed moderate immune reactions. Sitagliptin’s protective effect was also associated with reduced inflammation, and apoptotic markers. In conclusion, Sitagliptin is suggested to offer beneficial effects on the vascular health of cardiac blood vessels, thereby potentially reducing myocardial stress in diabetic patients.

## 1. Introduction

Diabetes mellitus (DM) is a global health problem characterized by chronic hyperglycemia, leading to various complications, including diabetic cardiomyopathy. An abnormal myocardial structure and performance are the hallmarks of diabetic cardiomyopathy, possibly without any associated cardiovascular risk factors [1,2]. It is characterized by ventricular dysfunction that occurs in diabetic patients in the absence of coronary atherosclerosis and/or hypertension [3]. A single pathophysiological pathway cannot explain the complex relationship between DM and cardiovascular complications. Both microvascular and macrovascular structures are affected by this complex interaction. Microvascular complications associated with diabetes include retinopathy, nephropathy, and neuropathy. However, macrovascular-associated complications can lead to atherosclerosis, coronary heart disease (CHD), stroke, and peripheral vascular disease [4].

Clinically, cardiac dysfunction in individuals with diabetes mellitus (DM) is often undetected in the early stages of the disease. Approximately 50% of asymptomatic patients with well-controlled DM exhibit some degree of cardiac dysfunction [3,5]. In DM, the impairment of the myocardium and cardiac functions, in the absence of other cardiac risk factors such as hypertension, valvular heart disease, and cardiovascular disease (CVD) like coronary artery disease contributes to the phenomenon known as diabetic cardiomyopathy [3]. This phenomenon is initially characterized by myocardial fibrosis, dysfunctional remodeling of the heart, and, eventually, HF. Several mechanisms were implicated in the development of diabetic cardiomyopathy, such as mitochondrial dysfunction, and oxidative stress, with inflammatory cytokines playing a key role in disease progression [6]. In this pro-inflammatory environment, the level of reactive oxygen species (ROS) generation increases, boosting cytokine production and circulation. The influx of cytokines, chemokines, and various leukocyte cells contributes to the development and progression of myocardial injury [3].

In diabetic heart disease, the death of cardiomyocytes, particularly through apoptosis, is a key feature of diabetic heart disease. Diabetic patients exhibit higher rates of cardiomyocyte apoptosis compared to non-diabetics, with various mechanisms implicated in this process [7,8]. Caspase-3, an apoptotic effector, is activated through both extrinsic (death receptor) and intrinsic (mitochondrial) pathways triggered by internal and external stress [9]. As a result of hyperglycemia, the overproduction of ROS production in the heart activates these apoptotic pathways, including the activation of caspase-3 [7].

Moreover, ROS directly stimulate nuclear factor kappa B (NF-κB) and trigger the release of inflammatory mediators [10]. Additionally, ROS inhibit the expression of the anti-apoptotic marker Bcl-2, while activating BAX, which facilitates cytochrome c release. This, in turn, activates the caspase cascade leading to apoptosis [11,12]. Indeed, the balance between pro-apoptotic BAX and anti-apoptotic Bcl-2 determines cell survival or death, and their regulation is crucial in protecting cardiac cells from apoptosis.

Another factor that contributes to cardiomyocytes apoptosis is the mammalian target of rapamycin (mTOR) pathway which regulates cell growth, survival, and metabolism, and is also implicated in diabetic cardiomyopathy, where its dysregulation contributes to pathological cardiac remodeling [13]. mTOR also influences apoptosis via several mechanisms. It activates p53, promoting BAX translocation to the mitochondrial membrane, and also stimulates the inositol-requiring kinase-1/C-jun NH2-terminal kinase pathway to inhibit Bcl-2, thereby increasing caspase-3 levels and inducing apoptosis [14].

In diabetes, the body shifts from using glucose to fatty acids (FAs) for energy production. However, fatty acid oxidation requires more oxygen than glucose oxidation, resulting in the production of excessive ROS. Unlike glucose, the oxidation of fatty acids requires a proportionally greater amount of oxygen to produce an equivalent amount of ATP [15]. Regrettably, FA oxidation due to increases in the generation of ROS has been implicated in apoptotic cell death [16].

In the diabetic heart, it is important to adopt strategies that promote angiogenesis, ensure a sufficient oxygen supply for metabolizing the excess FA, and prevent cell death caused by increased FA oxidation [17].

Vascular Endothelial Growth Factor (VEGF) is a critical protein involved in angiogenesis (the formation of new blood vessels). In cardiac cells, particularly under diabetic conditions, VEGF helps maintain blood flow to the myocardium (heart muscle) by promoting the growth of new capillaries. This process is crucial in diabetic hearts, which often suffer from poor oxygen delivery due to microvascular damage caused by hyperglycemia. VEGF Receptor 1 (VEGFR1), the protein known as “FLT-1”, is the receptor that binds to VEGF [18]. FLT-1 primarily binds VEGF-B and VEGF-A, modulating their activity. The balance between FLT1 and VEGFR2 is very important in regulating VEGF function in the cell, especially angiogenesis [19].

Sitagliptin is an oral anti-hyperglycemic agent and a dipeptidyl peptidase-4 inhibitor (DDP-IV). It was the first inhibitor used on a clinical basis and was approved in 2006 [20]. It works by prolonging the half-life of type I glucagon-like peptide (GLP-1), a hormone involved in glucose metabolism. In addition to its ability to control blood sugar levels, Sitagliptin has been shown to reduce cardiovascular complications related to diabetes. It exerts positive effects on the heart through its anti-inflammatory, anti-apoptotic, and antioxidant properties [21]. However, the impact of sitagliptin on angiogenesis proteins is not clear in cardiac blood vessels. The present study examined whether, in diabetic rats, the administration of DPP-IV inhibitor, an anti-hyperglycemic drug, affects the expression of mTOR, VEGF, and FLT-1 as part of their cardioprotective activity, in addition to their effect on inflammatory and apoptosis proteins, which, in turn, may contribute to the regeneration of blood vessels and reduce myocardial stress induced by hyperglycemia.

## 2. Materials and Methods

### 2.1. Experimental Animals

#### 2.1.1. Induction of Type I DM

Ten-week-old male Wistar rats (180–200) g were housed in specialized cages under controlled conditions with a temperature of 22 °C. The rats were obtained from the Animal Research Center/College of Pharmacy/King Saud University, KSA. They were provided with standard rat pellet chow, had free access to tap water ad libitum.

To establish a type-2 DM animal model, rats were fasted overnight and given a single intraperitoneal injection of Streptozotocin (STZ; Sigma-Aldrich, Burlington, MA, USA) at a dose of 55 mg/kg [22] prepared in 0.1 M citrate buffer (pH 4.5). The level of blood glucose was checked 72 h post-injection using a MEDISAFE MINI glucose meter (TERUMO Corporation, Tokyo, Japan), and rats showing glucose levels above 200 mg/dL were considered diabetic and selected for further experiments. To verify diabetes persistence, blood glucose was measured again 7 days after the STZ injection.

#### 2.1.2. Experimental Design

A total of 32 rats were divided into four groups, 8 rats for each as follows:

Control group: Normal rats received normal saline orally for 90 days.

Sitagliptin group: Normal rats were administered Sitagliptin (Merck & Co., Inc., Whitehouse, NJ, USA) at dose (100 mg/kg) [23] dissolved in saline via oral gavage for 90 days.

STZ group: Diabetic rats received normal saline by oral gavage for 90 days.

STZ+ Sitagliptin group: Diabetic rats received Sitagliptin (100 mg/kg/day) by oral gavage for 90 days.

Before the sacrifice day, the rats were fasted overnight (12 h), weighed, and measured. The blood glucose levels were measured using a MEDISAFE MINI blood glucose reader (Terumo company, Tokyo, Japan), anesthetized with gradually increasing concentrations of carbon dioxide (CO_2_), and sacrificed by decapitation. Blood was collected and processed to separate serum for glucose level, Troponin-I, CK-MB, and CRP analysis. Heart samples were excised and weighed. Part of the heart samples were homogenized in cold phosphate-buffered saline. The remaining tissues were prepared for histological and immune-histochemical processing.

### 2.2. Biochemical Analysis

#### 2.2.1. Heart Weight/Body Weight Ratio

The body weights of rats in all groups were recorded at the start of the experiment (Initial BW) and again before sacrifice (Final BW). Additionally, heart weights (HW) were measured post-dissection for all groups, and the heart weight to final body weight ratio (HW/Final BW) was calculated using the following formula:

HW/Final BW ratio = HW (g)/Final BW (g) ∗ 100(1)

#### 2.2.2. Determination of Serum Troponin I, and CK-MB and CRP Activity

Serum levels of troponin I (Cat# CSB-E08594r) and CK-MB (Cat# CSB-E14403r) and CRP (Cat# CSB-E07922r) activity levels were measured using ELISA kits (CUSABIO Technology LLC, Houston, TX, USA) according to the manufacturer’s instructions.

#### 2.2.3. Determination of Oxidative Stress

Lipid peroxidation, quantified as malondialdehyde (MDA), and reduced GSH levels were measured in heart homogenate according to the previous methods of Preuss et al. and Beutler et al., respectively [24,25]. The cardiac activities of Super Oxide Dismutase (SOD) were determined according to the protocols of Marklund and Marklund [26]. All chemicals for oxidative stress analysis were obtained by Sigma-Aldrich (USA).

#### 2.2.4. Determination of Inflammatory and Apoptotic Biomarkers

Levels of inflammatory biomarkers, including TNF-α (Cat# MBS700574) and IL-1β (Cat# MBS2530426) in cardiac homogenates using specific ELISA kits were measured. Also, the expression of BAX (Cat# CSB-EL002573RA), Caspase-3 (Cat# MBS018987), and Bcl2 (Cat# QT-ER0762) were estimated in cardiac tissues as pro-apoptotic and apoptotic biological markers, respectively.

### 2.3. Histological Study

Heart tissues were fixed, embedded in paraffin, and sectioned at 5 µm thickness. Sections were dewaxed, rehydrated, and stained with hematoxylin and eosin (H&E) to assess tissue structure and morphology of the heart or with Masson’s Trichrome stain for the detection of interstitial fibrosis. Stained heart sections were scanned and examined via light microscopy.

Additional heart tissue sections were prepared for immunohistochemical analysis targeting mTOR (Cat# sc-293133), NF-κB/p65 (Cat# sc-8008), VEGF (Cat# sc-7269), and FLT-1 (Cat# MBS126699). The procedure followed the guidelines provided with the Novolink Polymer Detection System (Cat# RE7280-K). Initially, tissue sections were immersed in 3% hydrogen peroxide for 5 min to block endogenous peroxidase activity. After a 10 min rinse in Tris-buffered saline (TBS; pH 7.6), a protein blocking reagent was supplied from Novocastra Laboratories Ltd. (Newcastle, UK) was applied for 5 min. The slides were then washed with TBS three times and incubated with a secondary antibody for ½ h. Following another TBS wash, the chromogenic substrate diaminobenzidine (DAB) was added, and the sections were counterstained with Mayer’s hematoxylin. Immunostaining was quantified using ImageJ software (version 1.54p, NIH, Bethesda, MD, USA), and results were expressed as a percentage relative to the control group.

### 2.4. Statistical Analysis

Our biochemical analysis data were evaluated by GraphPad Prism 9 (GraphPad Software, Inc., La Jolla, CA, USA). The comparisons were conducted through a one-way analysis of variance (ANOVA) followed by Tukey’s post hoc test. The results are presented as mean ± the standard error of the mean, and a *p*-value of less than 0.05 was considered statistically significant.

## 3. Results

### 3.1. Effects of Sitagliptin on DIABETIC Rats’ Body Weight and Blood Glucose Levels

**Figure 1:**
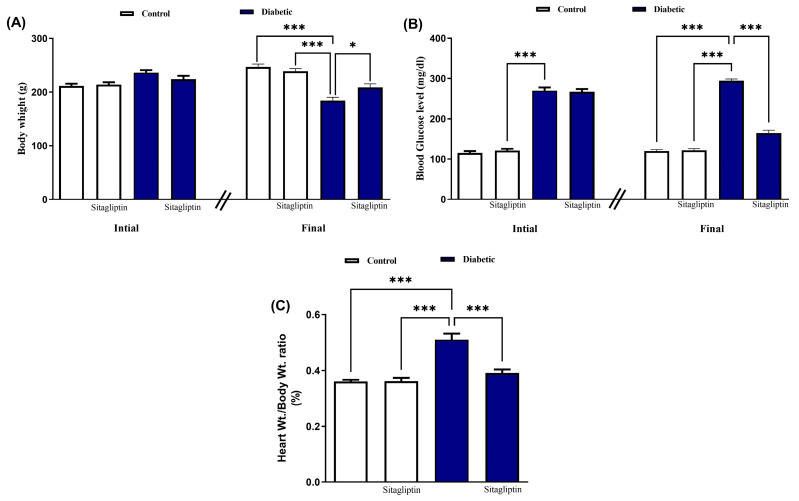
Sitagliptin ameliorated body weight (**A**), blood glucose level and (**B**) heart weight/body weight ratio (**C**) in STZ-diabetic rats. Data are mean ± SEM, (*n* = 8). (* *p* ≤ 0.05, and *** *p* ≤ 0.001). Abbreviations: SEM, standard error of the mean.

Figure 1A shows that the diabetic rats exhibited a significant reduction in body weight compared to the control group. Sitagliptin significantly mitigated this weight loss in diabetic rats over a 90-day supplementation period (*p* ≤ 0.01). In contrast, normal rats administered sitagliptin for 90 days showed no significant (*p*-value 0.99) change in body weight compared to the control group. The study revealed a significant increase in blood glucose levels in diabetic rats compared to controls. However, sitagliptin administration significantly reduced blood glucose levels in treated diabetic rats (*p* ≤ 0.001), with no impact on blood glucose levels in normal rats over the 90-day period (Figure 1B). Additionally, the heart weight to body weight ratio (HW/final BW) was significantly (*p* ≤ 0.001) elevated in diabetic rats compared to controls, an effect reversed by sitagliptin treatment (Figure 1C).

### 3.2. Effects of Sitagliptin on Cardiac Biomarkers

**Figure 2:**
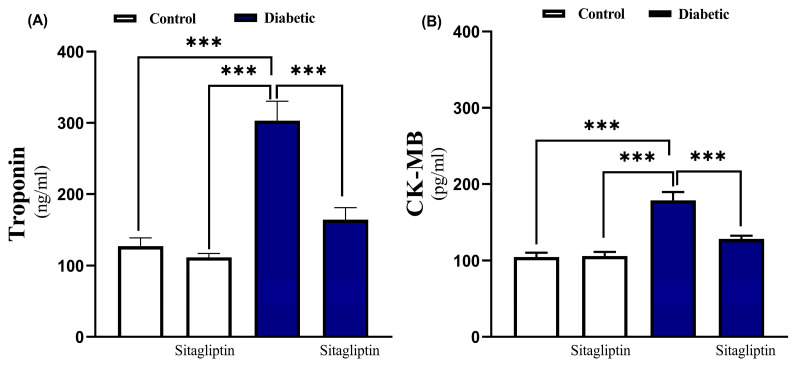
Sitagliptin improved serum level of cardiac biomarkers (**A**) Troponin1 and (**B**) CK-MB) in diabetic rats. Data are mean ± SEM, (*n* = 8). (*** *p* ≤ 0.001). Abbreviations: CK-MB, Creatine Kinase-MB; SEM, standard error of the mean.

To assess hyperglycemia-induced cardiac injury and the potential protective effects of sitagliptin, serum levels of troponin I and CK-MB were measured, alongside the histological examination. Diabetic rats showed significant (*p* ≤ 0.001) upregulation of serum troponin I and CK-MB compared to non-diabetic control. Conversely, sitagliptin-treated diabetic rats exhibited significant (*p* ≤ 0.001) improvements in serum troponin I and CK-MB levels compared to untreated diabetic rats, with no significant changes in normal rats treated with sitagliptin group (troponin I, *p* value 0.279), and (CK-MB, *p* value 0.984) (Figure 2).

### 3.3. Effects of Sitagliptin on Oxidative Stress

**Figure 3:**
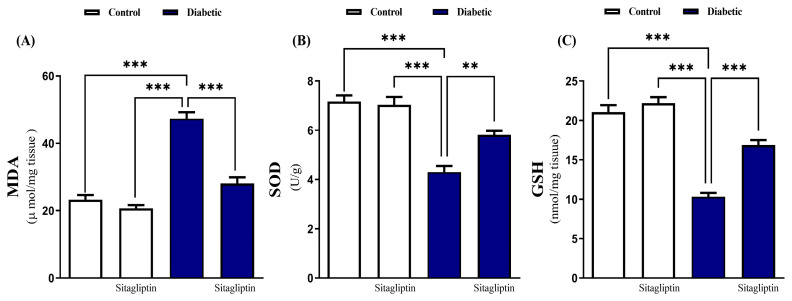
Sitagliptin ameliorated cardiac oxidative stress in diabetic rats. STZ increased MDA level in cardiac tissues (**A**), and decreased GSH level (**B**) and SOD activity (**C**); sitagliptin significantly improved oxidative stress. Data are mean ± SEM, (*n* = 8). (** *p* ≤ 0.01, and *** *p* ≤ 0.001). Abbreviations: MDA, malondialdehyde; SOD, superoxide dismutase; GSH, reduced glutathione; SEM, standard error of the mean.

Diabetic rats showed a significant (*p* ≤ 0.001) increase in cardiac MDA levels compared to normal rats (Figure 3A). Sitagliptin treatment significantly (*p* < 0.001) decreased cardiac MDA levels in diabetic rats, with no effect observed in normal rats. Furthermore, diabetic rats exhibited significant reductions in cardiac SOD activity (Figure 3B) and GSH levels (Figure 3C), which were significantly (*p* ≤ 0.01 and *p* ≤ 0.001, respectively) ameliorated by sitagliptin treatment, with no significant changes in MDA level (*p*-value 0.649), SOD activity (*p*-value 977), and GSH level (*p* value 0.676) as compared sitagliptin treated group and normal rats.

### 3.4. Histological Improvements

**Figure 4:**
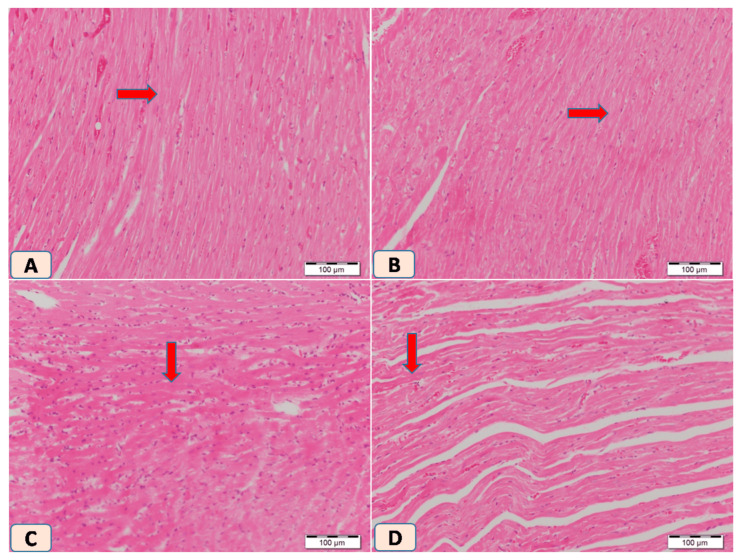
Photomicrographs of H&E-stained heart sections. (**A**) Control heart tissue showing normal myocardium (arrow). (**B**) Heart section from rats that received sitagliptin shows normal heart tissue (arrow). (**C**) Heart section from rats exposed to STZ showing multiple large patches of degenerated cardiac muscle cells and the cytoplasm is dark acidophilic with many pyknotic cells (arrow). (**D**) Heart section from rats exposed to STZ and treated with sitagliptin shows that most of the cardiac muscle cells were apparently normal, with few cells showing cytoplasmic degeneration (arrow). Magnification ×100.

Histopathological examination of H&E-stained cardiac sections from non-diabetic rats showed normal cardiac architecture. Diabetic rats, however, displayed multiple patches of cardiac degeneration, including loss of cardiomyocytes, inflammatory cellular infiltrations, and cytoplasmic degeneration (Figure 4C). In contrast, sitagliptin-treated diabetic rats showed a marked reduction in inflammatory infiltrations and normalization of cardiomyocyte cytoplasm and nuclei (Figure 4D).

**Figure 5:**
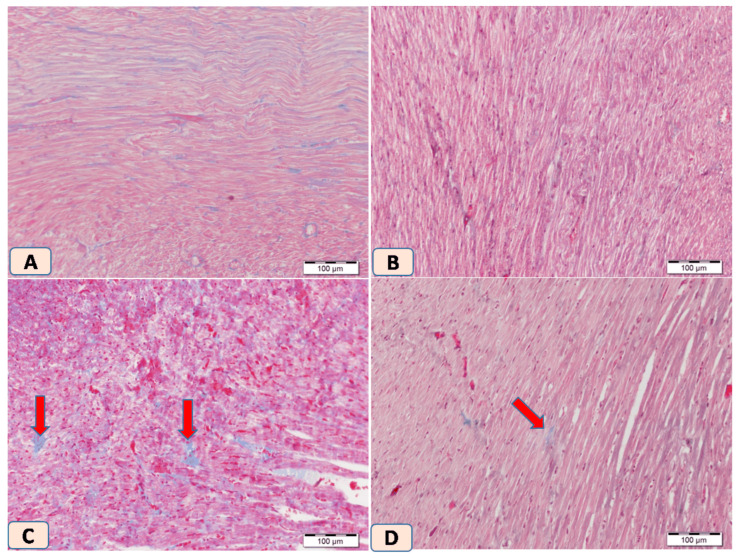
Photomicrographs of Masson trichrome-stained heart sections. (**A**) Control heart tissue showing normal amount and distribution of collagen mainly in the endomysium. (**B**) Heart section from rats that received sitagliptin also shows normal amounts of collagen between cardiac muscle fibers. (**C**) Heart section from rats exposed to STZ showing patches of collagen bundles between cardiac muscle cells (red arrows). (**D**) Heart section from rats exposed to STZ and treated with sitagliptin showing small patches of collagen (red arrows) and apparently normal distribution of collagen bundles that mimic the control one. Magnification ×100.

On the other hand, Masson trichrome-stained heart sections from control and sitagliptin groups showed normal amount and distribution of collagen mainly in the endomysium (Figure 5A,B). In dissimilarity, heart sections from rats exposed to STZ showed regeneration of multiple large patches of collagen bundles between cardiac muscle cells (Figure 5C). In contrast, heart sections from rats exposed to STZ and treated with sitagliptin showed apparently normal distribution of collagen bundles which mimic the control one (Figure 5D). Photomicrographs of H&E-stained heart sections showed normal myocardium in control heart tissue and as well in rats that received sitagliptin. Heart sections from rats exposed to STZ showed regeneration of multiple large patches of degenerated cardiac muscle cells; the cytoplasm is dark acidophilic with many pyknotic cells. Lastly, heart sections from rats exposed to STZ and treated with sitagliptin showed that most of the cardiac muscle cells were apparently normal with few cells showing cytoplasmic degeneration. Photomicrographs of Masson trichrome-stained heart sections in the control group showed heart tissue with normal amount and distribution of collagen mainly in the endomysium. Heart sections from rats that received sitagliptin also showed normal amounts of collagen between cardiac muscle fibers. Heart sections from rats exposed to STZ showed regeneration of multiple large patches of collagen bundles between cardiac muscle cells, and heart sections from rats exposed to STZ and treated with sitagliptin showed apparently normal distribution of collagen bundles which mimic the control one.

### 3.5. Cardiac Inflammation and Apoptosis Biomarkers

**Figure 6:**
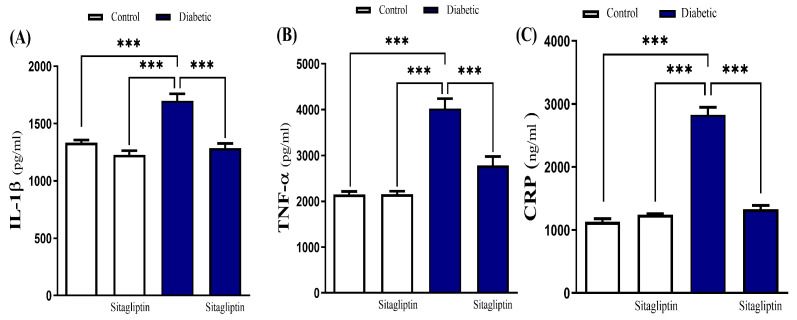
Sitagliptin downregulated cardiac inflammatory biomarkers IL-1β (**A**), TNF-α (**B**), and CRP (**C**) in diabetic rats. Data are mean ± SEM, (*n* = 8). (*** *p* < 0.001). Abbreviations: IL-1β, interleukin-1beta; TNF-α, Tumor necrosis factor-alpha; CRP, C-reactive protein; SEM, standard error of the mean.

**Figure 7:**
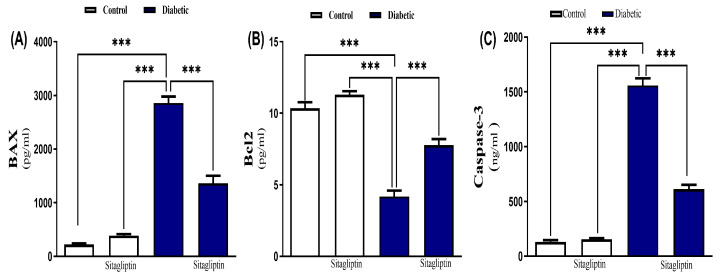
Sitagliptin improved apoptotic biomarkers in cardiac tissues, (**A**) BAX, (**B**) Bcl2, and (**C**) caspase-3 in STZ-induced diabetic rats. Data are mean ± SEM, (*n* = 8). (*** *p* < 0.001). Abbreviations: BAX, Bcl-2 associated X; Bcl-2, B-cell lymphoma 2; SEM, standard error of the mean.

Sitagliptin effect on diabetes-associated inflammation was assessed by measuring cardiac levels of IL-1β, TNF-α, and CRP, which were significantly elevated (*p* ≤ 0.001) in diabetic rats (Figure 6A–C). Sitagliptin treatment significantly reduced these inflammatory mediators. Additionally, STZ-induced diabetic rats exhibited significantly increased (*p* ≤ 0.001) cardiac levels of BAX and caspase-3, indicating apoptosis. This increase was significantly reduced by sitagliptin treatment (Figure 7A,C). Moreover, the STZ-diabetic group showed a significant inhibition of Bcl-2 levels compared to the control group. However, sitagliptin treatment led to a significant elevation in Bcl-2 levels compared to the diabetic group (Figure 7B).

### 3.6. Sitagliptin Upregulates mTOR/VEGF Signaling Pathways in Diabetic Rats

**Figure 8:**
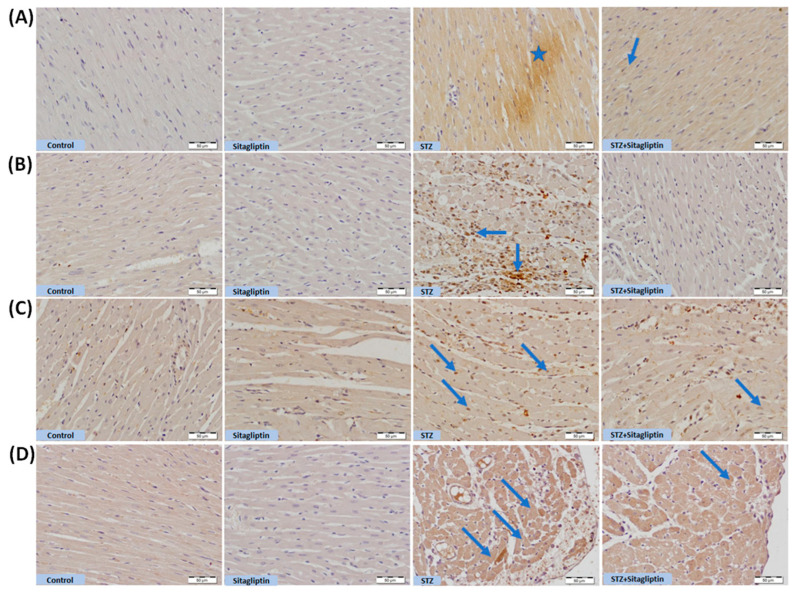
Photomicrographs of mTOR, NF-kB/P65, VEGF, and FLT-1 immune-stained heart sections. (**A**) mTOR, control heart tissue showing absence of immune reactivity. Heart section from rats that received sitagliptin also shows absent immune reaction. Heart section from rats exposed to STZ shows areas of strong positive immune reactivity (star). Heart section from rats exposed to STZ and treated with sitagliptin shows mild cytoplasmic immune reactivity (arrow). (**B**) NFkB-p65, control heart tissue shows absence of immune reactivity. Heart section from rats that received sitagliptin also shows no immune reaction. Heart section from rats exposed to STZ shows areas of strong positive immune-stained nuclei of myocardium (arrows), while heart section from rats exposed to STZ and treated with sitagliptin shows absence of immune reactivity. (**C**) Cardiac immune reactivity of VEGF in control heart tissue showing lack of immune reactivity. Heart section from rats that received sitagliptin also showed absence of immune reaction. Cardiac tissues section from rats exposed to STZ showed patches of myocardium cells with strong positive immune-stained nuclei, (arrows). On the other hand, heart section from rats exposed to STZ and treated with sitagliptin showed very few weak immune-stained nuclei (arrow). (**D**) Immune stating for FLT-1, control heart tissue showed lack of immune reactivity. Heart section from rats that received sitagliptin also showed absence of immune reaction, heart section from rats exposed to STZ showed patches of myocardium cells with strong positive immune-stained nuclei (arrows), and heart section from rats exposed to STZ and treated with sitagliptin showed few weak immune-stained myocardium cells (arrow). Magnification ×400, scale bar (50 µm).

IHC for mTOR staining indicates its hyperactivation in the cardiac tissue of diabetic rats as compared to the control (Figure 8A). Elevated NF-κB levels in STZ-diabetic tissues, represented by IHC staining (Figure 8B) compared to the control group, reflect increased inflammatory activity, exacerbating tissue damage and fibrosis. VEGF, a critical angiogenic factor, supports vascular development and repair. In diabetes, VEGF levels are often elevated in cardiac tissues as a compensatory response to vascular injury caused by hyperglycemia and endothelial dysfunction. Hyperactivation of the mTOR pathway in diabetic cardiac tissue increases VEGF production, contributing to abnormal angiogenesis. Increased VEGF staining, observed in this study (Figure 8C), indicates heightened angiogenic signaling, likely as an adaptive mechanism to address microvascular damage in the diabetic heart compared to control group.

FLT-1 serves as a regulator of VEGF signaling, while its soluble form (sFLT-1) acts as a decoy receptor, sequestering VEGF and preventing its interaction with VEGFR2, the primary mediator of angiogenesis. IHC staining demonstrated a significant increase in FLT-1 activity in STZ-diabetic rats compared to control rats (Figure 8D). Treatment with sitagliptin inhibitors significantly downregulated immune reactivity in mTOR, NFkB/P65, VEGF, and FLT-1 signaling pathways relative to untreated STZ-diabetic rats. Additionally, there was no significance in the sitagliptin treated group as compared to the normal control group.

## 4. Discussion

Different therapeutic strategies are available to minimize cardiovascular complications. They mainly act by regulating blood glucose and lipids rather than directly affecting the cardiac pathogenic pathways [27]. Sitagliptin is a DPP-IV inhibitor antidiabetic drug with evidence of potential cardioprotective activities [28]. However, further exploration of the exact cardioprotective mechanism is still needed [8]. This study aimed to explore the protective effect of sitagliptin on cardiomyopathy in the type I DM murine model and if its impact is associated with changes in the expression of mTOR, VEGF, and FLT-1.

The STZ-induced diabetic rat model is broadly used to study the effects of various treatments on cardiac complications in diabetes. STZ mainly damages insulin-producing β-cells, leading to the generation of ROS and impaired insulin secretion, all of which contribute to hyperglycemia. In this study, STZ-induced type-1 DM in treated rats was confirmed by persistent hyperglycemia. Additionally, STZ-treated rats experienced BW loss, likely due to the body’s reliance on lipids and proteins for energy, as it could not properly utilize glucose [17]. In the same context, our study revealed that sitagliptin significantly mitigated weight loss in diabetic rats over a 90-day supplementation period and reduced blood glucose significantly. Sitagliptin is recognized for its enhancement of GLP-1 secretion, which in turn improves glycemic control through the stimulation of insulin secretion and the inhibition of glucagon secretion [29]. This is achieved without the associated risks of hypoglycemia [30]. We revealed a significant reduction in blood glucose levels in diabetic rats treated with sitagliptin over the 90-day period. Our findings were similar to what had been recorded by many previous experiments [23] and clinical studies [31].

Alongside these metabolic disturbances, the diabetic rats also exhibited signs of cardiac dysfunction, with significantly higher levels of serum biomarkers such as Troponin I and CK-MB, in addition to HW/BW which increased considerably compared to control rats. These results were in accordance with previous studies [32,33]. Fortunately, STZ-induced cardiovascular effects in diabetic rats were reversed by sitagliptin treatment. Sitagliptin administrated in diabetic rats was accompanied by a decrease in both markers, i.e., CK-MB and Troponin I. Similar results were previously reported [23]. Additionally, cardiac histopathological changes in diabetic rats demonstrated myocardial cell hypertrophy, disarray of muscle fibers, and irregularities in the shape and size of nuclei. Treatment with sitagliptin mitigated these pathological changes. Bahriz and colleagues revealed the same results. They reported a significant improvement in myocardial cellular degeneration in diabetic rats treated with sitagliptin [34].

The pathogenesis of diabetic cardiomyopathy is complex, with many pathological mechanisms converging and overlapping together, culminating in its development and progression. It was found that oxidative stress and inflammation work in a synergistic manner, intensifying cellular damage [35]. Oxidative stress is widely recognized as a critical mediator in the development of complications associated with uncontrolled DM [36]. Hyperglycemia is often cited as the primary factor initiating the cascade of oxidative stress [35]. Consequently, the greater the efficacy of hypoglycemic drugs in controlling blood glucose levels, the more effective they are in preventing tissue oxidative stress complications [37]. Oxidative stress is claimed to reduce the level of antioxidants like SOD and GSH. The effects of sitagliptin as an antioxidant are reported through its ability to improve SOD activity and MDA levels [30]. These findings were confirmed in our research, with increased oxidative stress in diabetic rats evidenced by increased MDA, an indicator of lipid peroxidation, and reduced antioxidants (GSH and SOD). Sitagliptin could effectively improve the antioxidants/oxidant ratio and increase GSH and SOD significantly with a subsequent decrease in MDA.

Reports have indicated that hyperglycemia and ROS activate NF-κB/p65, a transcriptional regulator that can stimulate the expression of various inflammatory mediators. This induces an inflammatory status that culminates further myocardial damage [38]. Many previous studies mentioned that diabetic rats demonstrated an increase in myocardial NF-κB/p65, among many other inflammatory markers [39]. Additionally, numerous studies have indicated an increase in serum inflammatory biomarker levels in diabetic patients with cardiac hypertrophy [40]. In the current study, heart sections from rats exposed to STZ show positive NF-κB/p65 immunostaining, associated with a significant increase in other inflammatory markers, TNF-α, IL-1β, and CRP, compared to those of the control rats. Treatment with sitagliptin revealed a reduction in NF-κB/p65 immunoreactivity, in addition to significantly reducing other inflammatory mediators such as IL-1β, CRP, and TNF-α; our results were in accordance with a previously reported one [41]. Sitagliptin has also been recorded to enhance anti-inflammatory pathways in diabetic patients with high cardiovascular risk [42]. This effect was suggested to be through the inhibition of oxidative stress and also through GLP-1, which is known to have an anti-inflammatory function [43,44].

Both ROS and TNF-α can trigger cellular apoptosis. ROS can interact with nitric oxide to form peroxynitrite, a potent oxidant that damages DNA and activates apoptotic signaling. This leads to the inhibition of antiapoptotic Bcl-2 and the activation of Bax (a pro-apoptotic protein), with a subsequent release of cytochrome c, activation of caspase 3, DNA fragmentation, and ultimately, cell death [12]. This was evident in this study, with diabetic rats showing increased cardiac apoptotic markers; this was previously documented [32,45]. On the other hand, sitagliptin significantly attenuated apoptosis. Similar results demonstrated the positive effect of sitagliptin on the reduction in apoptosis in diabetic cardiac tissues [46]. mTOR was found to be linked to isoprenaline-induced cardiac hypertrophy pathogenesis (Zhang et al., 2015) [47], and several studies defined the hyperactivation of mTOR as a critical mediator of DM pathophysiology by enhancing insulin resistance and inflammation. Moreover, mTOR inhibitors like rapamycin have protective effects against diabetic cardiomyopathy [48,49]. It is worth mentioning that mTOR was found to be able to activate NF-κB and signal transducer and activator of transcription 3 (STAT3) through the formation of immunoproteasomes, both of which play a significant role in the pathogenesis of cardiac inflammation and fibrosis [47]. In the current study, mTOR immunoreactivity increased significantly in diabetic rats; this may be directly linked to the increased inflammatory markers. The hyperactivation of mTOR in diabetic cardiomyopathy was suggested to happen through a hyperglycemic state, which worsens the disease progression and contributes to the exacerbation of the inflammatory condition [48]. Treatment with sitagliptin ameliorated mTOR’s strong positive immune reactivity, which was shown in diabetic rats. This is the first study to document the inhibitory effect of sitagliptin on mTOR in a diabetic cardiomyopathy model. Sitagliptin inhibition of *mTOR* mRNA expression was previously reported in the aorta [50], and in the liver of STZ-induced diabetic rat model [22]. Furthermore, the increased mTOR and NF-κB/p65 activity in the diabetic group was accompanied by increased fibrous tissue deposition visualized by Masson Trichrome staining, and sitagliptin reduced the deposition [19], which can be explained in terms of its inhibitory effect on mTOR and NF-Κb/p65.

VEGF is a key growth factor that promotes vascular endothelial cell proliferation. mTOR is believed to upregulate the expression of *VEGF*, and thus, rapamycin inhibits it [51,52]. Our study supported this, where in diabetic rats, increased mTOR expression was associated with increased VEGF, while in rats treated with sitagliptin, reduced mTOR expression was associated with reduced VEGF expression. Sitagliptin reduced VEGF in cancer cells in a previous study [53]. To further understand the effect of sitagliptin on VEGF, the expression of FLT-1 was carried out. In the hearts of diabetic rats, FLT-1 expression was upregulated compared to the control group. Sitagliptin treatment was associated with reduced expression of FLT-1, compared to untreated STZ-diabetic rats. It is well-known that VEGF mainly binds to FLT-1 or VEGFR2, with FLT-1 being of less ability to induce angiogenesis, about one-tenths that of VEGFR2.

FLT-1 can bind tightly to VEGF but has a weak tyrosine kinase activity [54]. Thus, increased FLT-1 expression results in a consequent decrease in the binding of VEGF with VEGFR2. As the major angiogenic activity is induced by the binding of VEGF to VEGFR2 and the effect of FLT-1 in this regard is negligible [54,55], we, therefore, hypothesize that increased FLT-1 will lead to reduced VEGF activity as an angiogenic factor. Thus, although sitagliptin decreases VEGF expression, it does not necessarily reduce the angiogenic activity, as this is accompanied by reduced expression of FLT-1. This may explain the variability in reports about sitagliptin angiogenic activity in various tissues [55,56,57,58,59], due to its effect on the expression on different VEGFRs. This is the first study to link the cardioprotective effects of sitagliptin to its ability to inhibit VEGF and FLT-1 in a diabetic model. Further research is required to explore the complicated role of VEGFRs in the development of diabetic cardiomyopathy.

## 5. Conclusions

This study underscores the cardioprotective potential of sitagliptin in cardiomyopathy diabetic rats, with a focus on its influence on key protein pathways. This study further confirms the earlier reported sitagliptin’s antioxidant, anti-inflammatory, and anti-apoptotic effects. However, our study is the first to reveal that sitagliptin induces cardioprotective effects, at least in part, through modulation of the mTOR–VEGF–FLT-1 axis in diabetic cardiomyopathy. Indeed, our study introduces for the first time novel mechanistic insights of sitagliptin actions though demonstrating its inhibitory effects on FLT-1, a receptor linked to angiogenesis and vascular permeability, which may contribute to its protective mechanisms, warranting further investigation. While sitagliptin has been shown to influence mTOR in various tissues, such as the liver and aorta, this study is the first to show its effect on mTOR expression in cardiac tissue especially concerning diabetic cardiomyopathy. This finding is evolving our understanding of the potential of sitagliptin to protect the diabetic heart beyond glycemic regulation.

## Data Availability

The original contributions presented in this study are included in the article. Further inquiries can be directed to the corresponding author.
